# Differences in Dry Eye Questionnaire Symptoms in Two Different Modalities of Contact Lens Wear: Silicone-Hydrogel in Daily Wear Basis and Overnight Orthokeratology

**DOI:** 10.1155/2016/1242845

**Published:** 2016-08-31

**Authors:** Nery García-Porta, Laura Rico-del-Viejo, Alba Martin-Gil, Gonzalo Carracedo, Jesus Pintor, José Manuel González-Méijome

**Affiliations:** ^1^Clinical & Experimental Optometry Research Lab (CEORLab), Center of Physics (Optometry), School of Sciences, University of Minho, Gualtar, 4710-057 Braga, Portugal; ^2^Ocular Surface and Contact Lenses Research Group, University of Santiago de Compostela, Santiago de Compostela, 15782 A Coruña, Spain; ^3^Department of Optics II (Optometry and Vision), School of Optics, Universidad Complutense de Madrid, Madrid, Spain

## Abstract

*Purpose*. To compare the ocular surface symptoms and signs in an adult population of silicone-hydrogel (Si-Hy) contact lens (CL) wearers with another modality of CL wear, overnight orthokeratology (OK).* Materials and Methods*. This was a prospective and comparative study in which 31 myopic subjects were fitted with the same Si-Hy CL and 23 underwent OK treatment for 3 months. Dry eye questionnaire (DEQ) was filled in at the beginning of the study and then after 15 days, 1 month, and 3 months using each CL modality. The tear quality was evaluated with noninvasive tear break-up time. Tear production was measured with Schirmer test. Tear samples were collected with Schirmer strips being frozen to analyze the dinucleotide diadenosine tetraphosphate (Ap_4_A) concentration with High-Performance Liquid Chromatography (HPLC).* Results*. After refitting with ortho-k, a reduction in discomfort and dryness symptoms at the end of the day (*p* < 0.05, *χ* ^2^) was observed. No significant changes were observed in Ap_4_A concentration in any group. Bulbar redness, limbal redness, and conjunctival staining increased significantly in the Si-Hy group (*p* < 0.05, Kruskal–Wallis test).* Conclusion*. Discomfort and dryness symptoms at the end of the day are lower in the OK CL group than in the Si-Hy CL group.

## 1. Introduction

Nowadays, many people use contact lenses (CLs) and silicone-hydrogel (Si-Hy) CL in daily wear basis is the most commonly fitted modality of CL wear around the world [1]. However, soft CL wearers frequently report symptoms of discomfort and dryness, especially at the end of the day [2–4]; these symptoms are the most common reason of CL wear drop-out [5–8]. In the last years, the CL market has changed significantly with the introduction of new materials, designs, and care systems. In this regard, the use of silicone-hydrogel (Si-Hy) CLs may help reduce these symptoms [9, 10], as well as the use of some lens care solutions [11]. However, despite the attempts to solve these symptoms, dryness and discomfort remain the most frequent reason for CL wear discontinuation [5–8]. The prevalence of dryness and discomfort among soft CL wearers is higher than that in no CL wearers [7, 12, 13], having been estimated to be 50% [13–17]. In fact, dry eye (DE) related to CL wear has been included as a subtype of evaporative DE in the International Dry Eye Workshop in 2007 [18].

As it is seems clear that wearing soft CLs induces dryness and discomfort symptoms, avoiding CL wear during the day could be a good option in subjects who suffer from these symptoms. In this regard, orthokeratology (OK) is a technique used to reduce the refractive error temporarily. The refraction change is obtained by the programmed application of specially designed rigid gas permeable CLs [19]. In myopic patients, this procedure induces epithelial thinning of the central cornea and thickening in the mid-peripheral cornea, leading to a myopia reduction and improved unaided vision [20, 21]. With hyperpermeable lens materials, the OK CLs are worn during the night, while the subject is sleeping. Therefore, the subject has a good vision during the waking hours without using any type of CLs or spectacles.

Hence, the purpose of this study was to characterize and to compare the ocular surface symptoms using the dry eye questionnaire (DEQ) between two different modalities of CL wear. One group wore Si-Hy CLs and another one underwent OK treatment for three months.

On the other hand, some studies have shown poor correlation between the DE signs and symptoms [22, 23], so it is important to find objective biomarkers to help in the DE diagnoses. Previous studies have shown that several inflammatory cytokines show increased levels in DE patients [24–28]. Other relevant molecules are dinucleotides and Jesus Pintor Research Group found the presence of diadenosine polyphosphates in the tear film in 2002 [29]. The researchers also observed that the dinucleotide diadenosine tetraphosphate (Ap_4_A) concentration rises in patients with DE symptoms, with either normal or low tear production [30, 31], suggesting the possibility that this molecule could be an objective parameter for grading DE. Therefore, in collaboration with this group, a pilot study was performed comparing the subjective symptoms evaluated with the DEQ with the changes in Ap_4_A concentration in a small group of participants.

## 2. Materials and Methods

This was a prospective, nonrandomized, comparative study in which the ocular surface signs and symptoms were compared between a group fitted with a Si-Hy CL in daily wear basis and another one that underwent overnight OK treatment for 3 months. All participants were recruited at the Clinical and Experimental Optometry Research Lab (CEORLab) at the University of Minho (Portugal), with the sample being composed by staff and students from the University of Minho. All procedures conformed to the tenets of the Declaration of Helsinki. The Ethics Committee of the School of Sciences of the University of Minho (CEECUM) approved the study, and informed consent was obtained from each individual prior to the initiation of the study. All the participants had to satisfy the following inclusion and exclusion criteria (Table 1) to be eligible for the study.

At an initial visit, a comprehensive optometric examination has been done including determination of the refraction, visual acuity, and appropriateness to use contact lenses, and meeting the inclusion criteria. When necessary, an ophthalmologist evaluated the patient to establish whether he/she was appropriate candidate for contact lens wear. After that, 31 subjects were fitted with Si-Hy CL and 26 with OK CL, being the participants allocated in each group depending on the personal preferences and taking into account whether their ocular parameters allowed correcting the full myopia with the OK treatment or did not. Three participants in the OK group left the study during the first week of the OK treatment, so, for the statistical analysis, 31 Si-Hy CL wearers (6 men and 25 women) and 23 OK CL wearers (7 men and 16 women), who attended at least 1-month visit, were included.

All of the participants in the Si-Hy group were soft CL wearers but in the OK group 16 were soft CL wearers and 7 had not worn any type of CL before the study. All the participants were Caucasian and the characteristics of age, spherical equivalent (SE) refraction, visual acuity (VA), and corneal curvature are summarized in Table 2, with no statistically significant differences between both groups for any of these parameters.

The participants who were CL wearers were asked to discontinue their CL wear for 1 week before attending the baseline visit. Then, the scheduled visits were at 15 days and 1 month in the morning and at 3 months in the morning and afternoon for both groups. The participants were evaluated during the morning, maximum two hours after awaking, except for the 3-month afternoon visit, which was conducted at least 6 hours after the morning visit. In the OK group, more visits were scheduled at the beginning of the study to control the evolution of the OK treatment.

### 2.1. Contact Lenses (CLs)

The Si-Hy CL used was Biofinity^®^ (Comfilcon A, 48% water content, CooperVision) in daily wear basis and was replaced monthly. The maintenance solutions used were the following multipurpose disinfecting solutions (MPDSs): Synergi^®^ (Sauflon, UK), COMPLETE^®^ RevitaLens (Abbott Medical Optics, Santa Ana, CA), and OPTI-FREE^®^ PureMoist^®^ (Alcon, Fort Worth, TX). All of them include components to enhance the comfort of the CLs.

The OK CL used was Corneal Refractive Therapy^®^ (Paflucon D, Paragon CRT^®^). Paragon CRT Dual Axis was used in subjects with limbus-to-limbus corneal astigmatism. The initial CLs were fitted following the monograms of adaptation of the CRT manufacturer. If needed, some CL parameters were changed to obtain a full correction of the myopic refraction and, at the same time, a well centered CL. These CLs are replaced yearly, so if the first CL fit was successful, the participants used the same CLs during the 3 months. These CLs were used during the night, while the CL wearers were sleeping. The maintenance solution used was peroxide with saline solution to rinse the CLs before insertion. Moreover, they were instructed to use free preservative artificial tears to fill the CLs before being inserted in the eyes and to put a couple of drops in the eyes when they woke up, before removing their CLs, to avoid ocular surface damage when the patients removed the CLs.

### 2.2. Tests Performed

**Figure 1:**
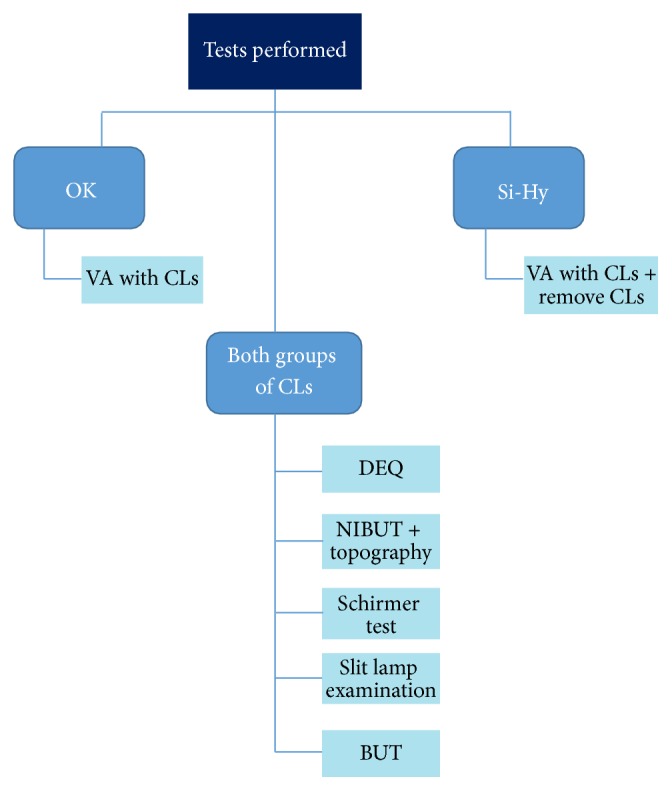
Order in which the tests were performed.

Figure 1 shows the order in which the tests were performed. After removing the CLs, the participants of the Si-Hy group waited 10 minutes before starting to perform the tests. Between each test, all the participants were asked to blink normally for at least 1 minute to recover the tear film stability.

### 2.3. Visual Acuity (VA)

The baseline VA was taken with the best spectacle correction and the VA in the follow-up visits was taken with CLs in the Si-Hy group and without CLs in the OK group. The VA was always measured with high contrast transilluminated ETDRS test under mesopic conditions.

### 2.4. Dry Eye Questionnaire (DEQ)

The long version of DEQ was used to evaluate the symptoms [15]. The DEQ contains questions about 8 symptoms: discomfort, dryness, sand sensation, burning sensation, itching, foreign body sensation, irritated eyes, and light sensitivity. For each symptom, frequency, morning and evening intensity, and how much the symptom bothers the subject are specified. For each question, the participants had five possible answers from 0 to 4. Regarding frequency, 0 means “never” and 4 means “constantly,” regarding intensity 0 means “nothing” and 4 means “very intensive,” and regarding bothersomeness 0 means “nothing” and 4 means “a lot.”

Although there is a new version of questionnaire available to use in CL wearers called Contact Lens Dry Eye Questionnaire (CLDEQ) [32], DEQ was used because it is not expected that the members of the OK group had symptoms, while they wore their CLs. Hence, all the participants answered the DEQ referring their symptoms during the day: in the Si-Hy group the symptoms happened while the subjects wore their CLs, and in the OK group the symptoms happened while they did not. The DEQ was always answered during the morning visits. As the Si-Hy group did not have any visit programmed after 15 days, the participants were asked to answer the DEQ at home and bring the questionnaire in the 1-month visit.

### 2.5. Tear Evaluation and Tear Collection

Tear quality was evaluated with noninvasive tear break-up time (NIBUT), using the Medmont topographer, and with the tear break-up time (BUT), using the slit lamp biomicroscopy, after instilling sodium fluorescein (NaFL) with a prepared strip of NaFL (BioGlo*™* Fluorescein Strips) wetted with a drop of saline solution. A yellow barrier filter (Wratten #12) was used to enhance the contrast. Both tests were assessed after asking the subjects to blink a couple times and the measurements were performed three times to obtain a more reliable value.

Schirmer test was used to measure the tear production. A TearFlo Schirmer strip (HUB Pharmaceuticals, USA) was placed in the temporal lower conjunctival sac of the OS and then the subjects were asked to keep their eyes closed for 5 minutes. The volume of tear was measured with the inked ruler of the Schirmer strip.

For the analysis of Ap_4_A concentration in the tears, the Schirmer strip used to measure the tear production was placed in 1.5 mL Eppendorf tube containing 500 *μ*L of ultrapure water. The samples were frozen until the high-pressure liquid chromatography (HPLC) analysis was performed. The method followed for Tear Preparation and HPLC Analysis was previously described by Carracedo et al. [33]. The samples analyzed were taken from 6 OK CL wearers and 5 Si-Hy CL wearers during the morning visits at baseline and at 3-month visits.

The normal tear volume is around 6 *μ*L and the mean tear secretion rate is 1.2 *μ*L per minute [34]. Therefore, at least 10-minute period was allowed since finishing the Schirmer test and before starting to measure the BUT. The ocular surface may not be totally restored from the Schirmer test after 10 minutes, so, to avoid differences between both groups, the test order was always the same.

### 2.6. Slit Lamp Evaluation

The ocular surface was examined using the slit lamp. To evaluate the conjunctival staining, bulbar, limbal, and lid redness, and tarsal roughness, the values were recorded according to the CCLRU grading scales [35]. The CCLRU scale has four images for each condition which increase in severity from 1, which means “very slight,” to 4, which means “severe.”

### 2.7. Statistical Analysis

The statistical analysis was conducted using SPSS v.21 (SPSS Inc., Chicago, IL, USA). Shapiro–Wilk test was used to evaluate the data distribution. Statistical significance was set at the level of *p* = 0.05 and the sample size was estimated for an 80% statistical power to detect differences of 1 score in the DEQ.

Chi-square (*χ* ^2^) test was used to evaluate the DEQ scores. ANOVA or Kruskal–Wallis tests were used to evaluate differences among all visits in the same group for the tear and ocular parameters, as well as VA. Bonferroni post hoc correction was used as post hoc correction. Independent sample* t*-test or Mann–Whitney test was used to evaluate differences between both groups of CLs in each visit. The tests used were chosen according to the data distribution. To avoid the duplication of the sample resulting from the interaction between both eyes from the same patient, only the left eye from each patient was used for statistical analysis.

Ap_4_A concentration was analyzed with nonparametric tests. Mann–Whitney test was used to detect differences between both groups and Wilcoxon test was used to analyze differences between baseline and 3-month visit in the same group.

## 3. Results

Three participants in the OK group discontinued during the first week of the OK treatment: two participants discontinued because they were not able to attend the scheduled visits and one case of discontinuation was due to a CL dislocation during the second night of OK CLs wear. The problem was solved successfully, without any impact on vision or corneal integrity. Furthermore, one subject from the OK group left the study after 1 month because her vision was not stable. There was one case of discontinuation during the third month in the Si-Hy group because one subject felt disappointed after breaking 2 CLs with the case of Synergi solution.

Regarding DEQ, no statistically significant differences between both groups were found at baseline for any symptom. With the use of the CLs, statistically significant differences were found only for discomfort and dryness. As Table 3 shows, discomfort and dryness symptoms at the end of the day were lower with the OK treatment, with the difference between both groups being statistically significant at 15 days for dryness and at 1 month for discomfort. The differences between both groups were maintained until the end of the study. Apart from the differences observed at the end of the day, statistically significant differences between both groups were found for frequency of dryness at 15 days and for bothersomeness of dryness at 1-month visit, with the DEQ scores being lower in the OK group. These differences were not maintained until the end of the study.

On the other hand, although no statistically significant differences were found between both groups, dryness during the first two hours of the day and bothersomeness of dryness improved with the use of Si-Hy CLs, and this change was statistically significant from baseline to 3-month visit (*p* < 0.05, Bonferroni post hoc correction).

Table 4 shows the changes in tear film stability and tear production for each group. There were no statistically significant differences in the Schirmer values, neither between both groups nor among the visits in each group of CLs. NIBUT values were higher in the OK group compared to Si-Hy group at baseline and continued being slightly higher during the study. The BUT values were slightly reduced at the 1-month visit in the Si-Hy but then came back to baseline values. When the differences from visit to visit were analyzed with Bonferroni post hoc correction, no statistically significant differences were found for BUT values, but a significant improvement from 1-month visit to 3-month visit was observed for NIBUT in the Si-Hy group.

Table 5 shows the slit lamp observations in both groups of CLs. Conjunctival staining, bulbar redness, and limbal redness increased with the use of Si-Hy CLs. Conjunctival staining increases significantly from visit to visit, with the highest values being observed at the 3-month afternoon visit. Bulbar redness and limbal redness increased significantly from baseline to the 1-month visit, and limbal redness also showed a significant increase at the 3-month from morning to afternoon. However, with the use of the OK CLs, limbal redness showed a significant reduction, with the differences between both groups being statistically significant at baseline and after 3 months of wearing the CLs. Lid roughness was significantly reduced with the use of the OK CLs, and the difference between both groups of CLs was statistically significant at 3-month visit.

Concerning Ap_4_A concentration in tear samples, no statistically significant differences were found between both groups, neither at the baseline nor at the 3-month visit (*p* > 0.05,* U* Mann–Whitney test). With the use of the OK CLs, no differences were observed, while with the use of Si-Hy CL, a slight decrease, although not statistically significant, was found (*p* > 0.05, Wilcoxon test).

## 4. Discussion

Dryness and discomfort symptoms, especially at the end of the day, are an important problem to solve for CL wearers [3, 4, 36]. In our sample, we observed a reduction in dryness and discomfort symptoms over time, even in the Si-Hy group. The deposits accumulated on frequent replacement CLs may lead to increased dryness and discomfort symptoms. In fact, the use of daily disposable CLs has been shown to be associated with enhanced comfort [37–39]. Considering that the CLs used in the present study are monthly replaced, the potential impact of deposit build-up on the CLs can be ruled out as a factor for dryness and discomfort. In a study of Wagner et al., it was observed that about 14% of subjects replaced their CLs only when there was a problem, rather than according to the manufacturer's recommendations [40]. The fact that we ensured that all the participants replaced their CLs accurately each month during the study may be an explanation for the improvement in the comfort scores. It may be also possible that the change of the CLs material or the change in the MPDS, or a combination of both things, helped improve these symptoms in the Si-Hy group.

Despite the improvement in the symptoms in both groups, the results of this study show that discomfort and dryness symptoms at the end of the day are more reduced with the use of OK CLs than with the use of Si-Hy CLs. In this regard, a case report published in 2013 showed that the OK treatment could be a good option for CL intolerant patients [41]. In the present study, the values obtained in the tear parameters do not justify this difference between both groups, neither the tear quality nor the tear production. Moreover, no changes in Ap_4_A concentration were observed, probably due to the small sample size. However, in the Si-Hy group, the highest scores in the DEQ were found at end of the day, and the highest values of conjunctival staining, bulbar redness, and limbal redness were observed at the 3-month afternoon visit. The increase in bulbar redness and limbal redness at the end of the day in the Si-Hy group seems to be related to the presence of the CL on the eye during several hours, despite the high oxygen permeability of the CL used. In a previous study, it was seen that the increases in bulbar redness and limbal redness with the three MPDSs used in this study were similar, supporting the idea that the ocular redness is related with the presence of the CL itself [42]. Moreover, this result agrees with other authors who observed that the evening peaks of redness coincide with the peak of CL awareness and dryness symptoms [2–4]. In addition, it was found that the conjunctival staining increased significantly in the Si-Hy group, while in the OK group it was kept constant. The most common type of conjunctival staining found in the Si-Hy group was a perilimbal staining, which may reflect an ocular response potentially related with CL dehydration or mechanical interaction of the CL with the ocular surface. According to previous studies, conjunctival staining could be related with the CL geometry, especially with the edge lens profile, and the material rigidity [43, 44]. In this regard, it has been seen that in DE symptomatic patients the number of Goblet cells is reduced [45]. These cells are the principal secretory cells in the conjunctival epithelia and their main function is to lubricate the ocular surface. It could be possible that the number of Goblet cells or their functions are altered during the Si-Hy CL wear and this would explain, in part, the higher DEQ scores in the Si-Hy group compared to the OK group. An additional explanation for the symptomatology reduction with the OK treatment might be found in the reduction of corneal sensitivity. This fact has been reported after one night of OK treatment [46]. If the corneal sensitivity reduction is maintained while the OK CLs are worn, this fact could explain in part the reduction in the dryness and discomfort symptoms. However, this factor may also be present in the Si-Hy group, since a reduction in mechanical sensitivity was observed in soft CL wearers at the end of the day [47]. Furthermore, it has been suggested that a lower CL dehydration rate might reduce dryness symptoms [48]. The dehydration rate is influenced by several factors, such as material features or environmental conditions [49, 50]. In this regard, an “in vitro” study published recently has shown that the Si-Hy CL used in this study is quite affected by the environmental conditions [50]. It could be possible that, using another Si-Hy CL with lower dehydration rate, the symptoms in the Si-Hy group were lower.

On the other hand, lid roughness decreased in the OK group, which may be related with the no solution delivery during the day from the OK CLs to the ocular surface. Moreover, the OK group was using peroxide solution and previous studies found that lid roughness is reduced with the use of peroxide, solution without preservatives [51, 52]. In this regard, during the study, both groups used different cleaning solutions that may have influence on the results obtained. The participants in the Si-Hy group used MPDSs that are the most common lens care solution used by soft CL wearers. However, the OK CLs wearers used peroxide solution, a very effective disinfecting solution that is rather important in yearly replacement CLs. The fact that the OK group used peroxide solution instead of MPDS may help reduce the symptoms in this group compared to the Si-Hy group, because a previous study found lower symptoms with peroxide solution than with MPDSs [53]. An additional factor that could affect the tarsal response may be the fact that with Si-Hy CLs the interaction between cornea and blink physiology is disturbed. Contrarily, in OK CL wear, the homeostasis of the ocular surface during the day is restored every day.

On the other hand, it is necessary to take into account the fact that all the participants in the Si-Hy group were soft CL wearers before entering in the study while in the OK group they were not, which could explain the fact that discomfort and dryness symptoms reached slightly higher scores (although not statistically significant) at the baseline in the Si-Hy groups. It is possible that if all the participants in the OK group were soft CL wearers, the symptomatology reduction with the OK treatment would be bigger. Moreover, as after sleeping with the OK CLs there is not enough tear between the OK CL and the cornea, the participants in the OK group were instructed to use artificial tears before removing their CLs to avoid damaging the cornea when the subjects removed the CLs. The artificial tears are present on the eye only for a few seconds and it is not expected that they have any effect over the dryness symptoms at the end of the day. These differences are consistent with the real differences existing in the clinic practice when these two types of CL are fitted. Another factor that may have influence on the results obtained is the fact that the Si-Hy subjects used their CLs during the autumn and winter and the OK group started the study during the spring and finished during the summer, when the weather is hotter and dryer. This fact should be favorable for better comfort with Si-Hy CLs than with OK CLs.

Several studies have compared the comfort between OK treatment and other CL wear modalities. Carracedo et al. showed that discomfort and dryness symptoms were lower with OK CLs compared to daily wear use of RGP CLs [33]. Lipson et al. compared hydrogel CLs in daily wear basis with OK CLs in a group of 65 people who used 8-week SCLS and 8-week OK CLs, worn in random order [54]. At the end of the study, 65% preferred to continue using OK CLs, and one of the reasons was to have less symptoms related with the CLs. Additionally, there are a couple of studies where the National Eye Institute Refractive Error Quality of Life Instrument (NEI-RQL-42) was used, and the results showed that the OK treatment is comparable to other modalities of myopic correction in terms of discomfort [55, 56].

In summary, despite the fact that discomfort and dryness symptoms at the end of the day are reduced in the Si-Hy group, the reduction with the OK CLs is larger. In this regard, the OK treatment could be an alternative to avoid drop-outs among CLs wearers who suffer from dryness and discomfort symptoms. However, more studies are needed to confirm that dryness and discomfort symptoms are reduced in long term. For further studies, it would be better that all the participants in the study used both types of CLs, starting in random order and changing the monthly replacement Si-Hy CL for the new daily disposable CLs available in the market. Apart from the subjective symptoms, it would be interesting trying to measure the ocular dryness with objective tests such as the osmolarity or taking more tear samples for analyzing DE biomarkers. Moreover, it is unknown if the corneal sensitivity is decreased while the OK CLs are worn. If the corneal sensitivity is more reduced in the OK treatment than in other CL modalities, this would explain, at least in part, the symptoms reduction but might have some negative effects.

## Figures and Tables

**Table 1:** Inclusion and exclusion criteria of the present study.

Inclusion criteria	Exclusion criteria
18 to 35 years of age Absence of ocular diseases including DE Flat keratometry between 40.0 and 45.0 D Refractive sphere between −1.00 and −6.00 D Able to understand and sign the consent form and to attend the scheduled visits	Not able to attend visits Symptomatic CL wearer Taking topical or systemic medication Astigmatism ≥ 1.00 D in the Si-Hy group and ≥1.75 D in the OK group Other clinically significant ocular findings compatible with inflammation History of ocular surgery

**Table 2:** Characteristics of the participants in the study.

	CL	Baseline
Age (years)	Si-Hy	22.97 ± 4.17
	OK	22.00 ± 2.45
	*p*	0.68^b^

SE refraction OS (D)	Si-Hy	−2.91 ± 1.31
	OK	−2.89 ± 1.17
	*p*	0.93^b^

K-flat OS (D)	Si-Hy	43.45 ± 1.25
	OK	43.45 ± 1.24
	*p*	0.99^a^

Monocular VA OS (LogMAR)	Si-Hy	−0.08 ± 0.06
	OK	−0.07 ± 0.06
	*p*	0.44^a^

Binocular VA (LogMAR)	Si-Hy	−0.15 ± 0.13
	OK	−0.13 ± 0.05
	*p*	0.52^a^

^a^ *t*-test independent; ^b^Mann–Whitney test; SE: spherical equivalent; OS: left eye; VA: visual acuity.

**Table 3:** Differences in the DEQ scores for each symptom in both groups of CL.

DEQ symptom	CL	Baseline	15 days	1 month	3 months
Frequency of discomfort	Si-Hy	1.48 ± 0.63	1.39 ± 0.88	1.45 ± 0.77	1.17 ± 0.64
	OK	1.09 ± 0.73	1.00 ± 0.77	0.96 ± 0.82	0.91 ± 0.81
	*p*	0.07^c^	0.53^c^	0.10^c^	0.06^c^

Discomfort during the first 2 hours of the day	Si-Hy	0.84 ± 0.97	1.10 ± 1.04	1.03 ± 0.87	0.73 ± 0.83
	OK	0.52 ± 0.59	0.86 ± 0.79	0.74 ± 0.91	0.82 ± 1.14
	*p*	0.17^c^	0.88^c^	0.35^c^	0.24^c^

Discomfort at the end of the day	Si-Hy	2.06 ± 1.24	1.84 ± 1.32	1.97 ± 1.11	1.67 ± 1.09
	OK	1.39 ± 0.99	1.09 ± 1.04	0.96 ± 0.98	0.77 ± 0.92
	*p*	0.24^c^	0.19^c^	0.02^c^	0.02^c^

Bothersomeness of discomfort	Si-Hy	1.93 ± 0.96	2.10 ± 1.28	1.84 ± 1.04	1.67 ± 1.15
	OK	1.30 ± 1.02	1.33 ± 1.11	1.13 ± 0.97	1.00 ± 1.02
	*p*	0.09^c^	0.05^c^	0.17^c^	0.07^c^

Frequency of dryness	Si-Hy	1.48 ± 0.72	1.61 ± 0.84	1.58 ± 0.81	1.43 ± 0.77
	OK	1.22 ± 0.60	1.00 ± 1.00	0.87 ± 0.87	1.27 ± 0.88
	*p*	0.49^c^	0.02^c^	0.02^c^	0.30^c^

Dryness during the first 2 hours of the day	Si-Hy	1.10 ± 1.01	0.84 ± 1.04	0.87 ± 1.06	0.57 ± 0.63
	OK	0.52 ± 0.59	0.76 ± 0.77	0.83 ± 1.27	1.00 ± 1.15
	*p*	0.06^c^	0.44^c^	0.30^c^	0.39^c^

Dryness at the end of the day	Si-Hy	2.23 ± 1.31	2.16 ± 1.29	2.10 ± 1.16	1.90 ± 1.18
	OK	1.61 ± 0.89	0.90 ± 1.04	1.00 ± 1.24	1.14 ± 1.08
	*p*	0.43^c^	0.01^c^	0.01^c^	0.01^c^

Bothersomeness of dryness	Si-Hy	2.19 ± 1.19	2.23 ± 1.52	2.19 ± 1.17	1.97 ± 1.24
	OK	1.56 ± 0.99	1.14 ± 1.15	1.04 ± 1.15	1.27 ± 0.98
	*p*	0.17^c^	0.16^c^	0.01^c^	0.06^c^

^c^Significance for Chi-square test (*χ* ^2^). Bold values in the same group for the same symptom show the visits in which statistically significant differences were found with Bonferroni post hoc correction.

**Table 4:** Differences in NIBUT, BUT, and Schirmer tests in both groups of CL. These results are only related to the left eyes.

Tear tests	CL	Baseline	1-month morning	3-month morning	3-month afternoon	*p*
NIBUT	Si-Hy	9.03 ± 3.10	7.60 ± 3.42	11.30 ± 6.80	9.60 ± 6.02	0.20^*∗*^
	OK	15.30 ± 9.01	10.61 ± 8.68	14.23 ± 10.90	12.23 ± 8.97	0.37^*∗*^
	*p*	0.01^b^	0.19^b^	0.43^b^	0.23^b^	

BUT	Si-Hy	7.17 ± 3.20	5.68 ± 3.40	7.85 ± 3.62	6.77 ± 2.01	0.03^*∗*^
	OK	8.74 ± 4.16	8.00 ± 6.26	7.36 ± 4.17	6.82 ± 2.94	0.38^*∗*^
	*p*	0.19^b^	0.05^b^	0.45^b^	0.98^b^	

Schirmer	Si-Hy	19.10 ± 11.62	18.93 ± 9.50	21.00 ± 11.79	19.03 ± 11.05	0.81^*∗*^
	OK	18.26 ± 10.58	21.09 ± 11.44	20.73 ± 11.10	18.32 ± 11.81	0.72^*∗*^
	*p*	0.85^b^	0.32^b^	0.85^b^	0.72^b^	

^*∗*^Friedman test. ^b^Mann–Whitney test. Bold values in the same group for the same symptom show the visits in which statistically significant differences were found with Bonferroni post hoc correction.

**Table 5:** Slit lamp observations in both groups of CL. These results are only related to the left eyes.

CCLRU signs	CL	Baseline	1-month morning	3-month morning	3-month afternoon	*p*
Corneal staining type	Si-Hy	0.47 ± 0.48	0.84 ± 0.82	0.79 ± 0.81	0.66 ± 0.77	0.27^*∗*^
	OK	0.33 ± 0.49	0.61 ± 0.78	0.64 ± 0.63	0.39 ± 0.53	0.34^*∗*^
	*p*	0.26^b^	0.21^b^	0.68^b^	0.22^b^	

Corneal staining depth	Si-Hy	0.48 ± 0.52	0.75 ± 0.72	0.64 ± 0.56	0.54 ± 0.58	0.22^*∗*^
	OK	0.31 ± 0.46	0.63 ± 0.77	0.62 ± 0.62	0.50 ± 0.58	0.38^*∗*^
	*p*	0.23^b^	0.41^b^	0.93^b^	0.74^b^	

Conjunctival staining	Si-Hy	0.21 ± 0.48	0.61 ± 0.71	1.55 ± 0.68	2.28 ± 0.78	<0.01^*∗*^
	OK	0.96 ± 0.64	0.92 ± 0.53	0.95 ± 0.81	0.95 ± 0.53	1.00^*∗*^
	*p*	<0.01^b^	0.05^b^	0.01^b^	<0.01^b^	

Bulbar redness	Si-Hy	1.47 ± 0.46	1.98 ± 0.52	1.91 ± 0.39	2.14 ± 0.35	<0.01^*∗*^
	OK	1.87 ± 0.48	1.67 ± 0.52	1.55 ± 0.40	1.69 ± 0.55	0.19^*∗*^
	*p*	0.07^b^	0.04^b^	<0.01^b^	<0.01^b^	

Limbal redness	Si-Hy	1.39 ± 0.46	1.78 ± 0.53	1.76 ± 0.33	1.95 ± 0.40	<0.01^*∗*^
	OK	1.66 ± 0.42	1.53 ± 0.49	1.38 ± 0.37	1.37 ± 0.45	0.04^*∗*^
	*p*	0.01^b^	0.10^b^	<0.01^b^	<0.01^b^	

Lid redness	Si-Hy	1.39 ± 0.44	1.46 ± 0.38	1.64 ± 0.36	1.54 ± 0.42	0.05^*∗*^
	OK	1.67 ± 0.58	1.54 ± 0.51	1.56 ± 0.52	—	0.68^*∗*^
	*p*	0.06^b^	0.66^b^	0.17^b^	—	

Lid roughness	Si-Hy	1.18 ± 0.51	1.06 ± 0.52	1.15 ± 0.56	1.14 ± 0.55	0.71^*∗*^
	OK	1.40 ± 0.77	1.11 ± 1.04	0.95 ± 1.06	—	0.02^*∗*^
	*p*	0.35^b^	0.54^b^	0.03^b^	—	

^*∗*^Kruskal–Wallis test. ^b^Mann–Whitney test. Bold values in the same group for the same symptom show the visits in which statistically significant differences were found with Bonferroni post hoc correction.

## References

[1] Morgan P. B., Woods C. A., Tranoudis I. G. (2016). International contact lens prescribing in 2015. *Contact Lens Spectrum*.

[2] Fonn D., Situ P., Simpson T. (1999). Hydrogel lens dehydration and subjective comfort and dryness ratings in symptomatic and asymptomatic contact lens wearers. *Optometry and Vision Science*.

[3] Begley C. G., Caffery B., Nichols K. K., Chalmers R. (2000). Responses of contact lens wearers to a dry eye survey. *Optometry and Vision Science*.

[4] Santodomingo-Rubido J., Barrado-Navascués E., Rubido-Crespo M.-J. (2010). Ocular surface comfort during the day assessed by instant reporting in different types of contact and non-contact lens wearers. *Eye and Contact Lens*.

[5] Dumbleton K., Woods C. A., Jones L. W., Fonn D. (2013). The impact of contemporary contact lenses on contact lens discontinuation. *Eye and Contact Lens*.

[6] Richdale K., Sinnott L. T., Skadahl E., Nichols J. J. (2007). Frequency of and factors associated with contact lens dissatisfaction and discontinuation. *Cornea*.

[7] Chalmers R. L., Begley C. G. (2006). Dryness symptoms among an unselected clinical population with and without contact lens wear. *Contact Lens and Anterior Eye*.

[8] Young G., Veys J., Pritchard N., Coleman S. (2002). A multi-centre study of lapsed contact lens wearers. *Ophthalmic and Physiological Optics*.

[9] Aakre B. M., Ystenaes A. E., Doughty M. J., Austrheim Ø., Westerfjell B., Lie M. T. (2004). A 6-month follow-up of successful refits from daily disposable soft contact lenses to continuous wear of high-Dk silicone-hydrogel lenses. *Ophthalmic and Physiological Optics*.

[10] Brennan N. A., Coles M. L. C., Comstock T. L., Levy B. (2002). A 1-year prospective clinical trial of balafilcon a (purevision) silicone-hydrogel contact lenses used on a 30-day continuous wear schedule. *Ophthalmology*.

[11] Campbell R., Kame G., Leach N., Paul M., White E., Zigler L. (2012). Clinical benefits of a new multipurpose disinfecting solution in silicone hydrogel and soft contact lens users. *Eye and Contact Lens*.

[12] González-Méijome J. M., Parafita M. A., Yebra-Pimentel E., Almeida J. B. (2007). Symptoms in a population of contact lens and noncontact lens wearers under different environmental conditions. *Optometry and Vision Science*.

[13] Guillon M., Maissa C. (2005). Dry eye symptomatology of soft contact lens wearers and nonwearers. *Optometry and Vision Science*.

[14] Doughty M. J., Fonn D., Richter D., Simpson T., Caffery B., Gordon K. (1997). A patient questionnaire approach to estimating the prevalence of dry eye symptoms in patients presenting to optometric practices across Canada. *Optometry and Vision Science*.

[15] Begley C. G., Chalmers R. L., Mitchell G. L. (2001). Characterization of ocular surface symptoms from optometric practices in North America. *Cornea*.

[16] Schafer J., Mitchell G. L., Chalmers R. L. (2007). The stability of dryness symptoms after refitting with silicone hydrogel contact lenses over 3 years. *Eye and Contact Lens*.

[17] Nichols J. J., Ziegler C., Mitchell G. L., Nichols K. K. (2005). Self-reported dry eye disease across refractive modalities. *Investigative Ophthalmology & Visual Science*.

[18] (2007). The definition and classification of dry eye disease: report of the Definition and Classification Subcommittee of the International Dry Eye WorkShop. *The Ocular Surface*.

[19] Swarbrick H. A. (2006). Orthokeratology review and update. *Clinical and Experimental Optometry*.

[20] Alharbi A., Swarbrick H. A. (2003). The effects of overnight orthokeratology lens wear on corneal thickness. *Investigative Ophthalmology and Visual Science*.

[21] Swarbrick H. A., Wong G., O'Leary D. J. (1998). Corneal response to orthokeratology. *Optometry and Vision Science*.

[22] Narayanan S., Miller W. L., Prager T. C. (2005). The diagnosis and characteristics of moderate dry eye in non-contact lens wearers. *Eye and Contact Lens*.

[23] Nichols K. K., Nichols J. J., Mitchell G. L. (2004). The lack of association between signs and symptoms in patients with dry eye disease. *Cornea*.

[24] Na K.-S., Mok J.-W., Kim J. Y., Rho C. R., Joo C.-K. (2012). Correlations between tear cytokines, chemokines, and soluble receptors and clinical severity of dry eye disease. *Investigative Ophthalmology and Visual Science*.

[25] Huang J.-F., Zhang Y., Rittenhouse K. D., Pickering E. H., McDowell M. T. (2012). Evaluations of tear protein markers in dry eye disease: repeatability of measurement and correlation with disease. *Investigative Ophthalmology and Visual Science*.

[26] Enríquez-de-Salamanca A., Castellanos E., Stern M. E. (2010). Tear cytokine and chemokine analysis and clinical correlations in evaporative-type dry eye disease. *Molecular Vision*.

[27] Lam H., Bleiden L., de Paiva C. S., Farley W., Stern M. E., Pflugfelder S. C. (2009). Tear cytokine profiles in dysfunctional tear syndrome. *American Journal of Ophthalmology*.

[28] Massingale M. L., Li X., Vallabhajosyula M., Chen D., Wei Y., Asbell P. A. (2009). Analysis of inflammatory cytokines in the tears of dry eye patients. *Cornea*.

[29] Pintor J., Carracedo G., Alonso M. C., Bautista A., Peral A. (2002). Presence of diadenosine polyphosphates in human tears. *Pflügers Archiv*.

[30] Peral A., Carracedo G., Acosta M. C., Gallar J., Pintor J. (2006). Increased levels of diadenosine polyphosphates in dry eye. *Investigative Ophthalmology & Visual Science*.

[31] Carracedo G., Peral A., Pintor J. (2010). Diadenosine polyphosphates in tears of Sjögren syndrome patients. *Investigative Ophthalmology & Visual Science*.

[32] Chalmers R. L., Begley C. G., Moody K., Hickson-Curran S. B. (2012). Contact Lens Dry Eye Questionnaire-8 (CLDEQ-8) and opinion of contact lens performance. *Optometry and Vision Science*.

[33] Carracedo G., González-Méijome J. M., Pintor J. (2012). Changes in diadenosine polyphosphates during alignment-fit and orthokeratology rigid gas permeable lens wear. *Investigative Ophthalmology & Visual Science*.

[34] Mishima S., Gasset A., Klyce S. D., Baum J. L. (1966). Determination of tear volume and tear flow. *Investigative Ophthalmology*.

[35] Terry R. L., Schnider C. M., Holden B. A. (1993). CCLRU standards for success of daily and extended wear contact lenses. *Optometry and Vision Science*.

[36] McMonnies C. W. (2013). Psychological and other mechanisms for end-of-day soft lens symptoms. *Optometry and Vision Science*.

[37] Cho P., Boost M. V. (2013). Daily disposable lenses: the better alternative. *Contact Lens and Anterior Eye*.

[38] Walker J., Young G., Hunt C., Henderson T. (2007). Multi-centre evaluation of two daily disposable contact lenses. *Contact Lens and Anterior Eye*.

[39] Fahmy M., Long B., Giles T., Wang C.-H. (2010). Comfort-enhanced daily disposable contact lens reduces symptoms among weekly/monthly wear patients. *Eye and Contact Lens*.

[40] Wagner H., Richdale K., Mitchell G. L. (2014). Age, behavior, environment, and health factors in the soft contact lens risk survey. *Optometry and Vision Science*.

[41] López-Lópeza M., Pelegrín-Sáncheza J. M., Sobrado-Calvob P., García-Ayusob D. (2011). Contact lens intolerance: refitting with dual axis lens for corneal refractive therapy. *Journal of Optometry*.

[42] García-Porta N., Rico-del-Viejo L., Ferreira-Neves H., Peixoto-de-Matos S. C., Queirós A., González-Méijome J. M. (2015). Performance of three multipurpose disinfecting solutions with a silicone hydrogel contact lens. *BioMed Research International*.

[43] Maïssa C., Guillon M., Garofalo R. J. (2012). Contact lens-induced circumlimbal staining in silicone hydrogel contact lenses worn on a daily wear basis. *Eye and Contact Lens*.

[44] Ozkan J., Ehrmann K., Meadows D., Lally J., Holden B., De la Jara P. L. (2013). Lens parameter changes under in vitro and ex vivo conditions and their effect on the conjunctiva. *Contact Lens and Anterior Eye*.

[45] Zuazo F., López-Ponce D., Salinas-Toro D. (2014). Conjunctival impression cytology in patients with normal and impaired OSDI scores. *Archivos de la Sociedad Espanola de Oftalmologia*.

[46] Lum E., Golebiowski B., Gunn R., Babhoota M., Swarbrick H. (2013). Corneal sensitivity with contact lenses of different mechanical properties. *Optometry and Vision Science*.

[47] Martín-Montañez V., López-de la Rosa A., López-Miguel A., Pinto-Fraga J., González-Méijome J. M., González-García M. J. (2015). End-of-day dryness, corneal sensitivity and blink rate in contact lens wearers. *Contact Lens and Anterior Eye*.

[48] Morgan P. B., Efron N. (2000). Hydrogel contact lens ageing. *CLAO Journal*.

[49] Brennan N. A., Efron N., Bruce A. S., Duldig D. I., Russo N. J. (1988). Dehydration of hydrogel lenses: environmental influences during normal wear. *Optometry and Vision Science*.

[50] Martín-Montañez V., López-Miguel A., Arroyo C. (2014). Influence of environmental factors in the in vitro dehydration of hydrogel and silicone hydrogel contact lenses. *Journal of Biomedical Materials Research Part B: Applied Biomaterials*.

[51] Sorbara L., Jones L., Williams-Lyn D. (2009). Contact lens induced papillary conjunctivitis with silicone hydrogel lenses. *Contact Lens and Anterior Eye*.

[52] Maissa C., Guillon M., Wong S., Lane A., Patel T., Garafalo R. Silicone hydrogel contact lenses effects on eyelid tissues: lens care influence.

[53] Keir N., Woods C. A., Dumbleton K., Jones L. (2010). Clinical performance of different care systems with silicone hydrogel contact lenses. *Contact Lens and Anterior Eye*.

[54] Lipson M. J., Sugar A., Musch D. C. (2005). Overnight corneal reshaping versus soft disposable contact lenses: vision-related quality-of-life differences from a randomized clinical trial. *Optometry and Vision Science*.

[55] Ritchey E. R., Barr J. T., Mitchell G. L. (2005). The Comparison of Overnight Lens Modalities (COLM) study. *Eye and Contact Lens*.

[56] Queirós A., Villa-Collar C., Gutiérrez A. R., Jorge J., González-Méijome J. M. (2012). Quality of life of myopic subjects with different methods of visual correction using the NEI RQL-42 questionnaire. *Eye and Contact Lens*.

